# Broadband S-Band Stripline Circulators: Design, Fabrication, and High-Power Characterization

**DOI:** 10.3390/mi17010063

**Published:** 2025-12-31

**Authors:** Aslihan Caglar, Hamid Torpi, Umit Kaya

**Affiliations:** 1 Department of Electronics and Communication Engineering, Yildiz Technical University, Istanbul 34220, Turkey; torpi@yildiz.edu.tr; 2 ETAS Elektron Teknolojileri Inc., Ankara 06378, Turkey; bilgi@elektronteknolojileri.com; 3 Institute of Accelerator Technologies, Ankara University, Ankara 06830, Turkey

**Keywords:** stripline circulator, broadband, ferrite materials, S-band, below-resonance operation, high-power RF measurements, magnetic field mapping, impedance matching, non-reciprocal devices

## Abstract

A stripline-type circulator is essential for the initial low-power characterization of vacuum electron devices such as magnetrons, enabling accurate measurements of startup behavior, oscillation frequency, and mode structure while minimizing reflections and protecting diagnostic equipment. In this study, two broadband S-band stripline circulator prototypes operating in the 2–4 GHz and 3–4 GHz bands were designed, fabricated, and experimentally characterized. A unified design methodology was implemented by using the same ferrite material and coupling angle in both structures, providing procurement simplicity, cost reduction, and technological standardization. This approach also enabled a direct assessment of how bandwidth variations influence circulator behavior. The design goals targeted a transmission efficiency above 90%, isolation exceeding 15 dB, and a voltage standing-wave ratio (VSWR) of 1.2:1. Experimental evaluations, including magnetic field mapping, low-power S-parameter measurements, and high-power tests, confirmed that both prototypes satisfy these specifications, consistently achieving at least 90% transmission across their respective operating bands. Additionally, a comparative analysis between a locally fabricated ferrite and a commercial ferrite sample was conducted, revealing the influence of material properties on transmission stability and high-power behavior. The results demonstrate that broadband stripline circulators employing a common ferrite material can be adapted to different S-band applications, offering a practical, cost-effective, and reliable solution for RF systems.

## 1. Introduction

Circulators are passive components that form an essential part of modern radio frequency (RF) and microwave systems, including telecommunications, radar, RF amplifiers, and communication networks. A circulator, typically consisting of three ports, allows for unidirectional transmission of electromagnetic waves [1]. This functionality is induced by the nonreciprocal behavior of anisotropic magnetic materials, such as ferrites, when biased by an externally applied static magnetic field.

**Figure 1:**
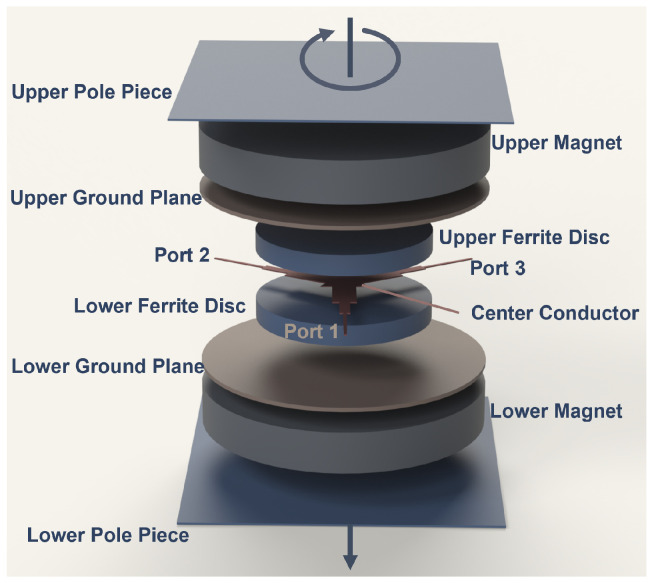
Fundamental structure and components of a three-port circulator.

The fundamental structure and components of a circulator are illustrated schematically in Figure 1. Under a perpendicular magnetic field applied to the center ferrite disk, an electromagnetic wave entering from one port splits into clockwise and counterclockwise rotating components. Due to the symmetry of the configuration, these components interfere in such a way that the signal couples only to the next port while being isolated from the remaining one. When three transmission lines (e.g., stripline, microstrip, or waveguide) are connected at 120 ° intervals, this mechanism establishes one-way power transfer in the directions port1→port2, port2→port3 and port3→port1 [1,2].

The operation of a circulator can be expressed by a 3×3 scattering matrix (S) that defines the relationship between three input/output ports. In the ideal case, this matrix provides complete transmission (0 dB) to the subsequent port while ensuring isolation that approaches infinity, i.e., $|S_{ij}|\to 0$, at the remaining port. When the third port is terminated with a matched RF load that fully absorbs the incoming signal, the circulator behaves as an isolator, transmitting power from port1→port2 while effectively suppressing reverse transmission from port2→port1 [1,2].

Vacuum electron devices, such as magnetrons, can be characterized at early-stage low-power tests using stripline circulators to suppress reflections and make network-analyzer measurements possible. By providing a matched load through its isolated port, the stripline circulator ensures stable oscillation, minimizes frequency pulling and mode hopping, and enables accurate and repeatable measurements. In low-power tests, impedance characteristics and quality factors derived from resonance curves can be evaluated, while weak external excitation allows the study of injection-pulling and injection-locking behaviors. In addition, the effects of anode voltage and magnetic field strength on oscillation stability can be systematically investigated.

In accordance with these requirements, this paper presents the design, fabrication, and experimental characterization of two broadband S-band stripline circulators operating in the 2–4 GHz and 3–4 GHz bands. Hereafter, the circulator designed for the 2–4 GHz band is denoted as Prototype-1, whereas the circulator designed for the 3–4 GHz band is denoted as Prototype-2. Table 1 presents the design parameters corresponding to both structures, where the same ferrite material was implemented due to being easy to procure, cost-effective, and contributing to technology standardization. During the construction and characterization stage, two different ferrite and dielectric material sources were tested: a commercial supplier from USA and a low-cost, academic laboratory alternative from Turkey.

## 2. Literature Review

The design of circulators involves various scientific approaches, including the selection of the ferrite material and calculation of electromagnetic wave propagation and scattering parameters, and ensuring impedance matching. This is an inherently complex process that requires the simultaneous optimization of numerous physical parameters. Since there is no universal closed-form design equation, many studies in the literature rely on empirical methods, analytical modeling, and experimental validation [3]. Key studies in the literature are summarized below.

Bosma [4] introduced the first theoretical foundations of the Y-type stripline circulator and provided a mathematical analysis of the behavior of the electromagnetic wave in ferrite materials under an applied magnetic field. He demonstrated that for effective circulation in the above-resonance region, the applied magnetic field must be at least four times greater than the resonance field. Fay and Comstock [5] developed a model to describe the below-resonance behavior of circulators. They showed that the mode splitting resulting from the separation of overlapping resonance modes in ferrite at low magnetic field levels directly influences key performance parameters such as isolation, insertion loss, and bandwidth. This influence intensifies as the ferrite approaches saturation magnetization. Taken together, these studies indicate that the magnitude of the applied magnetic field is the dominant factor determining circulator performance in both above and below-resonance conditions and that different design strategies are required to achieve optimum performance at different field levels.

**Figure 2:**
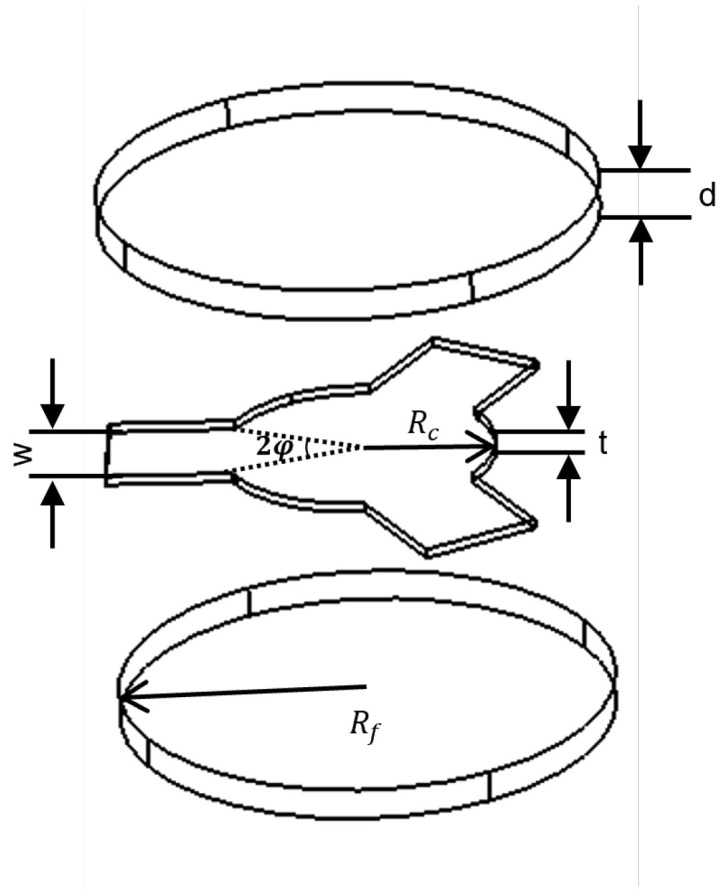
Schematic representation of the Y-type center conductor and ferrite geometry.

The first study combining theoretical modeling with experimental validation for broadband stripline circulators operating in the below-resonance region was carried out by Wu and Rosenbaum [6]. This approach, known as continuous tracking, demonstrated that ideal circulation can be achieved at kR≈1.2 with a coupling angle of φ≈0.5 rad. The geometrical definitions of kR and φ are shown in Figure 2. Their results further indicated that employing a wide coupling angle together with a small center conductor radius offers clear advantages, particularly by simplifying fabrication and enhancing bandwidth performance.

Following this model, Helszajn [7] analyzed the circulation conditions of stripline circulators using the eigennetwork approach and derived a new expression for the quality factor. He demonstrated that when the ferrite is saturated, results consistent with the *Q* factor are obtained at κ/μ=0.67 and φ=0.522 rad. The continuous tracking theory was further extended by introducing semi-tracking solutions by shifting the coupling angle (φ) and magnetization (κ/μ) values [8]. By incorporating a two-stage quarter-wave impedance transformer, low VSWR values were achieved across an octave bandwidth. The study presented both theoretical formulations and practical design tables, and using these data, a circulator operating in the 2.6–5.2 GHz band was designed, manufactured and experimentally validated, showing excellent agreement with theoretical predictions.

The influence of ferrite material properties on circulation performance has been demonstrated through experimental studies across a wide range of frequencies. Jaiswal and Pradeepkumar [9] developed a stripline isolator operating in the 3.3–4.3 GHz band using Yttrium–Aluminum (Y–Al) ferrite, achieving an insertion loss below 0.36 dB and isolation exceeding 23 dB. Ghadiya et al. [10] reported a Yttrium–Gadolinium (Y–Gd) garnet ferrite-based design operating in the 10.5–13.5 GHz band, which maintained insertion loss below 0.5 dB and demonstrated thermal stability within the temperature range of −10 °C to +60 °C. Schloemann and Blight [11] developed circulators employing Yttrium Iron Garnet (YIG) disks in the 2.8–10.2 GHz band and Lithium Ferrite (Li-ferrite) disks in the 5.8–18 GHz band, showing that a two-octave bandwidth can be achieved by reducing insertion losses and improving magnetic field homogeneity.

The performance of these structures is determined not only by the ferrite type and resonator geometry but also by the uniformity of the applied magnetic field. Ayasli [12] employed the integral equation method to accurately model the electromagnetic field distribution within the ferrite resonator and demonstrated that the incorporation of boundary conditions into analytical solutions resulted in excellent agreement between theoretical predictions and experimental results.

In modern applications, high-power and broadband circulator designs are realized by combining closed-form solutions with simulation-based analyses. Khim et al. [13] introduced equations that directly calculate ferrite parameters ($4\pi M_{s}$, ΔH), Y-junction geometry, and impedance matching networks, allowing designers to define circulators operating in below-resonance (BR) and above-resonance (AR) modes without resorting to trial-and-error. The study also provided a detailed investigation of power handling capability, demonstrating through HFSS-based simulations that prototypes operating in the 6–18 GHz band can withstand up to 870 W peak power and 13 W average power, while outlining strategies to optimize both thermal and electrical limits.

Ali [14] addressed the limitations of classical methods for analyzing stripline Y-junction circulators by accounting for ferrite disk thickness and the actual field distribution. A newly proposed Gaussian Field Distribution (GFD) boundary condition assumes that the fields around the ports spread according to Gaussian profiles. On the basis of this formulation, closed-form solutions for input impedance and field distribution were derived. The study reported excellent agreement with 3D electromagnetic simulations (CST), with errors below 5%, and demonstrated that frequency shifts and demagnetization effects associated with increased ferrite thickness can be accurately modeled.

The reviewed studies indicate that achieving successful broadband below-resonance stripline circulator design requires the simultaneous optimization of impedance matching, ferrite selection, resonator sizing, and uniform magnetic field distribution. Accordingly, the design approach presented in this work was developed on the basis of theoretical and experimental models reported in the literature, and both analytical and numerical evaluations were performed for a broadband structure operating in the below-resonance region.

## 3. Design, Simulation, and Fabrication of Stripline Circulators

This section describes the design of broadband stripline circulators. First, analytical methods are described to determine the ferrite parameters, resonator dimensions, and impedance matching conditions. Next, electromagnetic simulations based on the derived analytical equations are described to validate and optimize the designs. Finally, the fabrication of prototypes is described, following the simulations, in order to realize the proposed structures.

### 3.1. Analytical Methods

The design parameters were determined analytically based on an extensive review of the literature. The first step involved calculating the ferrite saturation magnetization ($4\pi M_{s}$), which directly influences the center frequency, bandwidth, and insertion loss. Several approaches for calculating $M_{s}$ have been reported [9,13]; in this work, the appropriate range was obtained using Equation (1).

(1) $$\frac{f}{3\gamma }\leq 4\pi M_{s}\leq \frac{3f}{4\gamma },\;\frac{f}{2\gamma }-H_{a}\leq 4\pi M_{s}\leq \frac{4f}{5\gamma }-H_{a}$$

Here, *f* is the center frequency, γ is the gyromagnetic ratio (2.8 MHz/Oe), and $H_{a}$ denotes the anisotropy field varying within 0–100 Oe. For circulators operating in the below-resonance mode, the internal magnetic field is assumed to be $H_{dc}=0$, meaning that the ferrite is not fully magnetically saturated [3]. Under this condition, the relative permeability is approximated as μ=1, and the effective magnetic permeability of the ferrite, $\mu_{\mathrm{eff}}$, can be expressed as:

(2) $$\mu_{\mathrm{eff}}=\frac{\mu^{2}-\kappa^{2}}{\mu }=1-\frac{\gamma^{2}{\left(4\pi M_{s}\right)}^{2}}{f^{2}}$$

where μ and κ represent the real and imaginary parts of the permeability tensor, respectively. The obtained $\mu_{\mathrm{eff}}$ serves as a key parameter linking ferrite properties to resonant behavior and provides the basis for determining the radius of the ferrite disk, which is given by Equation (3).

(3) $$R_{f}=\frac{kR}{2\pi f\sqrt{\mu_{\mathrm{eff}}\mu_{0}\varepsilon_{r}\varepsilon_{0}}}=\frac{c(kR)}{2\pi f\sqrt{\mu_{\mathrm{eff}}\varepsilon_{r}}}$$

According to Equation (3), *c* is the speed of light, $\mu_{0}$ and $\varepsilon_{0}$ are the permeability and permittivity of free space, $\varepsilon_{r}$ is the dielectric constant of the ferrite, and kR=1.84 is a shape-dependent constant obtained from the solution of the Bessel function. This relation indicates that the radius of the ferrite disk depends on both the intrinsic properties of the ferrite and the operating frequency. After determining $R_{f}$, the next step is to specify the center conductor geometry to achieve proper coupling within the Y-junction configuration, as illustrated in Figure 2, which shows the geometry of a typical stripline circulator including the center conductor and the ferrite disk.

The geometry of the center conductor can be designed in triangular, hexagonal, or star forms. These variations strongly affect parameters such as the resonant frequency, the susceptance slope ($B^{\prime }$), and the conductance ($G_{r}$) of the center conductor, although having only a limited influence on the loaded quality factor ($Q_{L}$) [2]. The conductance of the circulator at the center frequency is expressed as a function of the wave admittance in Equation (4) [5]:

(4) $$G_{r}=\frac{Y_{\mathrm{eff}}\left|\kappa /\mu \right|}{\sin\varphi }$$

with φ denoting coupling angle and κ/μ representing the mode-splitting ratio. The wave admittance ($Y_{\mathrm{eff}}$), determined by the dielectric and magnetic properties of the ferrite, is given in Equation (5).

(5) $$Y_{\mathrm{eff}}=\sqrt{\frac{\varepsilon_{0}\varepsilon_{r}}{\mu_{0}\mu_{\mathrm{eff}}}}$$

In this expression, $\varepsilon_{0}$, $\varepsilon_{r}$, and $\mu_{\mathrm{eff}}$ correspond to the vacuum permittivity, the relative dielectric constant, and the effective magnetic permeability, respectively. The loaded quality factor ($Q_{L}$) characterizes the relation between the energy stored in the ferrite and the power transferred to the strip lines [2,5]. A low $Q_{L}$ provides a wider isolation bandwidth, while a high $Q_{L}$ reduces insertion loss and facilitates more efficient power transfer. This relation is defined as follows:

(6) $$Q_{L}=\frac{1.48\omega R_{f}^{2}\varepsilon_{r}\varepsilon_{0}}{G_{r}d}$$

where *d* is the thickness of the ferrite and ω is the angular frequency. To estimate the insertion loss of a below-resonance circulator, Fay and Comstock [5] defined the unloaded quality factor, which is given in Equation (7).

(7) $$Q_{u}=\frac{1}{\left(\frac{\gamma^{2}4\pi M_{s}\Delta H}{2\omega^{2}}+\tan\delta \right)}$$

In this formula, tanδ denotes the dielectric loss tangent of the ferrite, and ΔH corresponds to the linewidth. Using the calculated $Q_{u}$, the insertion loss can be obtained from Equation (8).

(8) $$\mathrm{Insertion}\;\mathrm{Loss}\;\left(\mathrm{dB}\right)=10\log_{10}\left(1-\frac{Q_{L}}{Q_{u}}\right)$$

Thickness of the ferrite is a fundamental design parameter and is often approximated as λ/16 (free-space wavelength), as suggested by Simon [15]. However, this value should not be regarded as a strict limitation for stripline circulators, since variations in thickness can be compensated by modifying either the center conductor geometry or the stripline width [4]. For example, if a thinner ferrite is required in a disk-type resonator, switching to a corner-coupled triangular geometry can reduce the thickness to nearly one-third, whereas implementing a side-coupled triangular geometry can increase it by a factor of three. As expressed in Equation (6), the ferrite thickness (*d*) is inversely related to both the susceptance slope ($B^{\prime }$) and the resonator input conductance ($G_{r}$). As illustrated in Figure 2, the width (*W*) of the center conductor guiding the electromagnetic wave in the circulator is directly proportional to the coupling angle (φ). In the Y-junction configuration, the three transmission lines are symmetrically connected to the center conductor at 120 ° intervals [2]. The stripline width is defined in Equation (9) as a function of the coupling angle and the center conductor radius $R_{c}$.

(9) $$W=2R_{c}\sin\varphi$$

**Figure 3:**
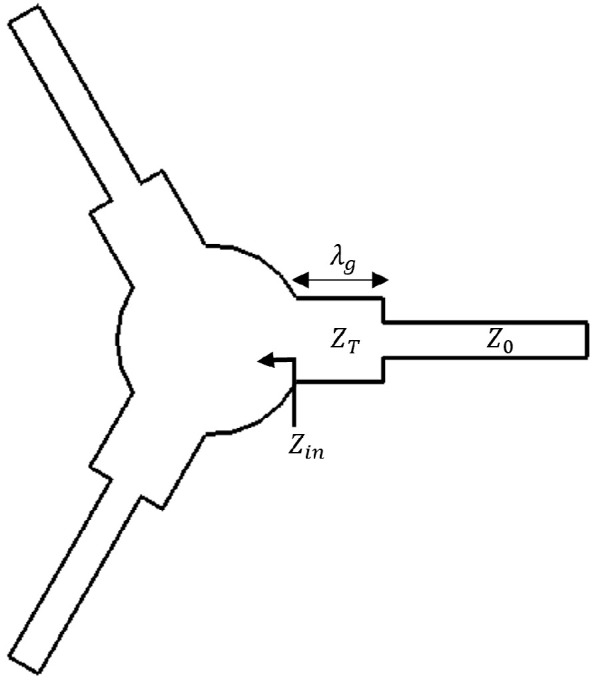
Geometry of a single-stage quarter-wave impedance transformer.

Smaller coupling angles are particularly advantageous for broadband operation. Wu and Rosenbaum [6] demonstrated that reducing the coupling angle from φ=0.3 to φ=0.1 under weak coupling conditions increases the bandwidth. These findings are supported by analyses based on the continuous tracking technique and are consistent with the measurement results reported in [7,8,15]. In addition, according to the study of Jaiswal and Pradeepkumar [9], the center conductor diameter should be chosen to be approximately 80% of the resonator disk diameter for broadband applications. The bandwidth, for cases where the circulator junction impedance is not matched to the system characteristic impedance (typically $Z_{0}=50\;\Omega$), was defined by Bosma [4] in Equation (10):

(10) $$BW=2.9\frac{\kappa }{\mu }\Gamma_{\max}$$

where $\Gamma_{\max}$ is the maximum voltage reflection coefficient within the band and is directly related to VSWR. As indicated by this relation, the bandwidth depends on the resonance frequency splitting caused by two counter-rotating modes formed within the ferrite resonator. The magnitude of this splitting is proportional to the gyromagnetic ratio κ/μ. In the absence of impedance matching, the achievable bandwidth remains limited. To overcome this limitation, quarter-wave impedance transformers are commonly employed to match the input impedance of the center conductor to the system impedance. These transformers convert the resonator impedance to the system characteristic impedance, thereby allowing broader bandwidths [2,9]. A single-stage transformer is illustrated in Figure 3, and its transformation relation is given in Equation (11).

(11) $$Z_{T}^{2}=Z_{0}Z_{\mathrm{in}}$$

Here, $Z_{T}$ denotes the characteristic impedance of the transformer line, and $Z_{\mathrm{in}}$ is the input impedance to be matched. The electrical length of the transformer is chosen as $\lambda_{g}/4$ at the design frequency, where $\lambda_{g}$ is the guided wavelength.

In this context, single-stage transformed structures remain narrowband. For broadband applications, multi-stage or more complex transformer topologies (e.g., Chebyshev, Binomial) are preferred. A detailed analysis of these design methods is discussed by Pozar [1]. For a circulator to operate, an external magnetic field must be applied to initiate the gyration of electrons in the ferrite. It is essential that this field remain homogeneous and uniformly distributed over the ferrite; otherwise, some regions may not be fully magnetized, leading to degraded performance and increased insertion loss [3]. In below-resonance circulators, the internal magnetic field of a not-yet-saturated ferrite is commonly assumed to be zero. However, to avoid low-field losses, the applied field must be strong enough to drive the material into saturation. The optimum internal magnetic field $H_{\mathrm{in}}$ is defined in Equation (12) as the combined effect of the externally applied field $H_{dc}$, the anisotropy field $H_{a}$, and the demagnetization field $H_{d}$.

(12) $$H_{\mathrm{in}}=H_{dc}+H_{a}-H_{d}$$

Here, the demagnetization field is defined as $H_{d}=N_{z}4\pi M_{s}$, where $N_{z}$ is the demagnetization factor determined by the ferrite geometry and the orientation of the applied field. Its value lies between 0 and 1, and for disk-shaped ferrite structures, $N_{z}\approx 0.88$ has been reported in the literature [16]. The anisotropy field $H_{a}$ accounts for the effect of randomly oriented magnetic dipoles within the ferrite and is typically chosen in the range of 0–100 Oe to represent these effects and compensate for experimental uncertainties.

### 3.2. Practical Method in Simulations

In this part of the study, the parameters calculated by analytical methods were validated and optimized through full-wave electromagnetic simulations performed with the Frequency Domain Solver of CST-MWS [17]. The design process started by evaluating Equation (1) at the center frequency and continued with the determination of the optimum saturation magnetization. The ranges obtained from this calculation are presented in Table 2.

A ferrite material consistent with the properties in Table 2 was investigated by surveying several manufacturers. During this process, parameters such as saturation magnetization, resonance linewidth, and dielectric constant were considered. Based on these evaluations, the aluminum-doped AL800 ferrite from TCI [18] was selected for use in both prototypes due to its low-loss characteristics. The material properties are summarized in Table 3.

The next step of the design was to select the dielectric material surrounding the ferrite, for which the guidelines reported in the literature were also considered. In accordance with the study by Elhanafy et al. [19], the design frequency should be set approximately 5% higher than the center frequency, and the relative permittivity of the dielectric should be about 60% lower than that of the ferrite material. Accordingly, the K9 dielectric material from TCI [18], with $\varepsilon_{r}=9$ and tanδ<0.0002, was implemented. In both designs, the dielectric thickness was set equal to the ferrite thickness to eliminate discontinuities caused by height differences and to simplify assembly. Based on these recommendations, the ferrite disk radius was calculated using Equations (2) and (3), and these values are summarized in Table 4.

Following the determination of ferrite and dielectric properties, it is equally important to evaluate the geometric ratios that govern the resonant behavior of the structure. Linkhart [3], as expressed in Equations (13), defined the ratio of the radius of the air or insulating region surrounding the ferrite ($R_{d}$) to the ferrite radius ($R_{f}$) as a key parameter for keeping the resonant frequencies outside the operating band. He further indicated that, for disk-type ferrites, selecting the center conductor radius ($R_{c}$) within a specific range of ratios provides suitable performance for broadband operation.

(13) $$1.3\leq \frac{R_{d}}{R_{f}}\leq 1.7,\;1.5\leq \frac{R_{f}}{R_{c}}\leq 2$$

The bandwidth performance of circulators is primarily determined by the impedance matching between the center conductor junction and the standard 50 Ω ports. This matching is generally obtained by using one- or two-section quarter-wavelength impedance transformers. As proved by Linkhart [3], based on VSWR value between minimum and maximum specified in the operating band, the coupling impedance ($Z_{r}=1/G_{r}$) should be in the range of 12.5–25 Ω. Variations in this parameter affect the loaded quality factor and ferrite thickness. In this study, the synthesis method described in [3] was implemented to determine the network parameters, with the equations solved iteratively to ensure accuracy and efficiency. The resulting values for two prototypes operating in different frequency bands, derived using the specified VSWR constraints, are presented in Table 5.

The thickness (*t*) of the center conductor is a key geometric parameter in the design of the stripline circulator, directly influencing the distribution of the electromagnetic field, the characteristic impedance and the density of RF current. Variations in conductor thickness can significantly influence impedance values and therefore limit system performance [1]. In this study, the thickness of the center conductor was fixed at 0.3 mm to ensure the desired impedance match and to simplify fabrication.

Based on these design choices, the analytically obtained parameters for the circulator designs are summarized in Table 6. As standard products with ferrite radius and thickness values exactly matching the calculated results were not available on the market, custom fabrication was considered but not preferred due to the high costs and long lead times associated with overseas suppliers. Instead, another AL800-type ferrite (diameter: 15.9 mm, thickness: 2.54 mm), which provided the closest physical properties to the target values, was procured from TCI [18]. For the dielectric layer, the electrical properties and geometric dimensions of the required K9 material [18] were specified, and the material was produced by a local manufacturer, Solak Laboratory (Solak Lab., (Kupfer Advanced Materials Technologies, Istanbul, Turkey)) [20]. In addition, to evaluate the performance of domestically produced ferrite materials, an AL800-type [20] ferrite sample with the same dimensions was manufactured locally by Solak Laboratory.

**Figure 4:**
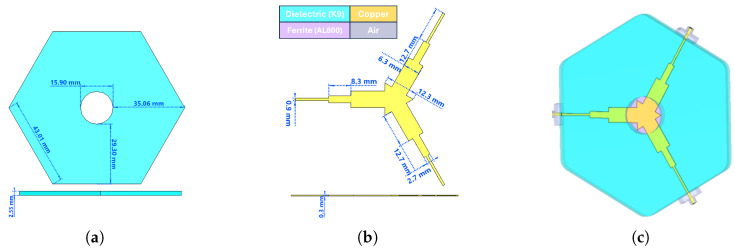
2–4 GHz stripline circulator: (**a**) dielectric dimensions, (**b**) center conductor dimensions, (**c**) three-dimensional design view.

**Figure 5:**
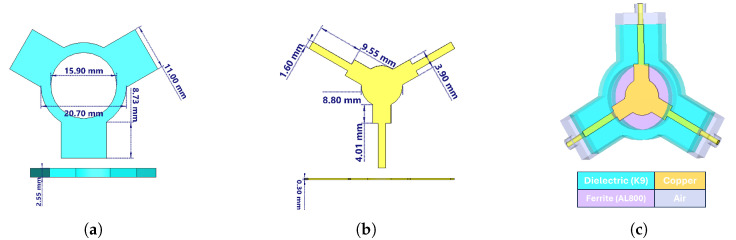
3–4 GHz stripline circulator: (**a**) dielectric dimensions, (**b**) center conductor dimensions, (**c**) three-dimensional design view.

The coupling impedance $Z_{r}$ calculated for prototype-1 in Table 5 was only 4.6 Ω, which is considerably lower than expected. To address this limitation, the disk-type center conductor was redesigned into a side-coupled triangular geometry, increasing the impedance to 13.85 Ω—a nearly threefold improvement [3]. Since the design aimed to cover a full octave bandwidth, three Chebyshev impedance transformers were employed instead of two, as this approach provides improved matching and wider bandwidth. The theoretical background and design methodology of this transformer topology are described in detail in [1]. The updated analytical results are summarized in Table 7. Using these parameters, circulators for both frequency bands were modeled in CST Microwave Studio, and frequency-domain simulations were carried out [17]. Figure 4 and Figure 5 present the three-dimensional designs of the prototype-1 and prototype-2 circulators, respectively.

**Figure 6:**
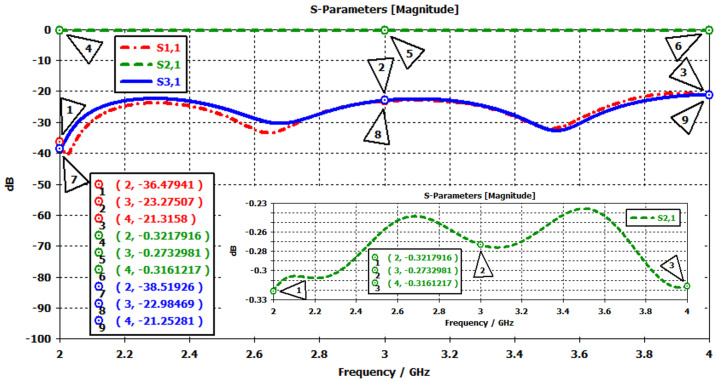
S-parameter results of the stripline circulator operating in the 2–4 GHz band obtained via the Frequency Solver in CST-MWS.

**Figure 7:**
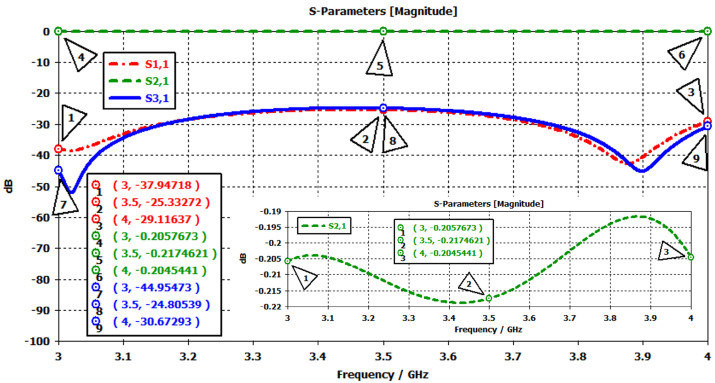
S-parameter results of the stripline circulator operating in the 3–4 GHz band obtained via the Frequency Solver in CST-MWS.

As seen in Figure 4c and Figure 5c, transition sections were designed between the coaxial line (SMA) and the stripline in both prototypes. While the coaxial line supports a pure TEM mode, the stripline operates in a quasi-TEM mode. Therefore, the transition was introduced to enable a smooth conversion from the coaxial TEM mode to the quasi-TEM stripline mode while also compensating for the reactive part of the impedance. The total transition length was set equal to the SMA connector pin length (5 mm), and the remaining dimensions were optimized to reach the desired performance. As summarized in Table 7, the lengths of the quarter-wave transformers were slightly extended to achieve proper impedance transformation. The S-parameter results obtained after these optimizations are shown in Figure 6 and Figure 7.

Prototype-1 was simulated using the parameter values listed in Table 7. The corresponding results are shown in Figure 6. The targeted frequency range is clearly covered, as indicated by the $S_{11}$ trace (red curve), which remains between −21 and −36 dB, confirming good input matching. The insertion loss ($S_{21}$, green curve) is approximately −0.3 dB across the band, demonstrating low-loss performance. The isolation ($S_{31}$, blue curve) varies between −21 and −38 dB. These results confirm that prototype-1 achieves broadband operation with low insertion loss, strong input matching, and acceptable isolation levels across the band.

With a similar approach, one can observe that the other prototype also provides the desired results, as declared in Table 1. The corresponding S-parameter results are shown in Figure 7. The $S_{11}$ trace (red curve) remains well below −20 dB across the entire band, with values of −38 dB at 3 GHz, −25 dB at 3.5 GHz, and −29 dB at 4 GHz, indicating good input matching. The insertion loss ($S_{21}$, green curve) is nearly constant at around −0.2 dB, confirming efficient low-loss transmission. The isolation characteristic ($S_{31}$, blue curve) varies between −25 and −45 dB, reaching a maximum of −45 dB at 3 GHz and maintaining −30 dB at 4 GHz.

**Figure 8:**
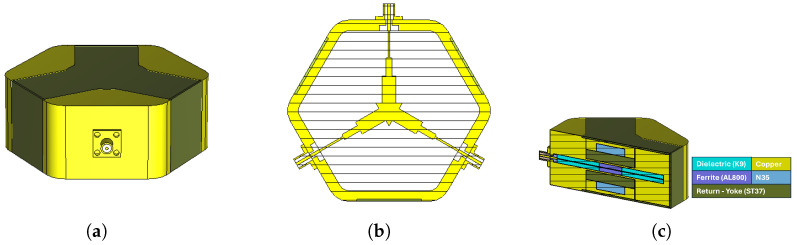
Simulation model of the 2–4 GHz stripline circulator for magnetic field analysis: (**a**) full view, (**b**) side view, (**c**) cross-sectional view.

**Figure 9:**
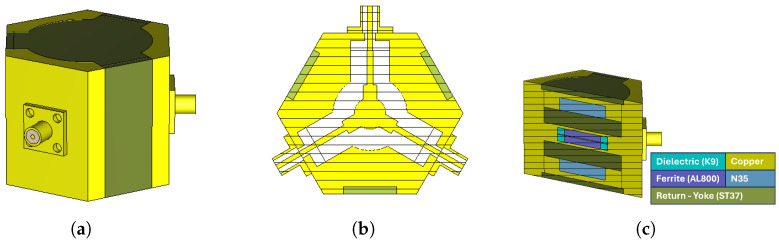
Simulation model of the 3–4 GHz stripline circulator for magnetic field analysis: (**a**) full view, (**b**) side view, (**c**) cross-sectional view.

In frequency-domain simulations, an external static magnetic field was applied to enable the ferrite material to exhibit circulation properties. For the prototype-1 and prototype-2 designs, the required magnetic field values were determined as 160 Gauss and 215 Gauss, respectively. The corresponding total magnetic field intensities, according to the relation $4\pi M_{s}+H_{dc}$, are 960 Gauss and 1015 Gauss. To generate the external magnetic field, either permanent magnets or solenoids can be used. In this study, permanent magnets were employed, and the magnetic field simulations were carried out using the CST EM solver [17]. The three-dimensional models of prototype-1 and prototype-2 are shown in Figure 8 and Figure 9, respectively. In both designs, permanent magnets with the same dimensions and specifications were used. The properties of the permanent magnets employed are summarized in Table 8.

**Figure 10:**
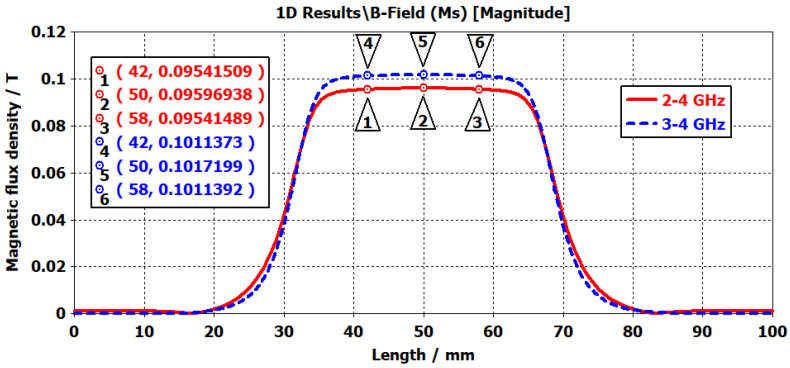
Simulated magnetic flux density distributions for the 2–4 GHz circulator (solid red line) and the 3–4 GHz circulator (dashed blue line).

In ferrite materials, achieving high transmission and isolation requires a homogeneous magnetic field distribution. To obtain a more uniform magnetic field over the ferrite, soft steel (ST37) plates with relatively high magnetic permeability were incorporated into the design. The dimensions of these plates were selected as ⌀34.4/6 mm for prototype-1 and ⌀34/6.2 mm for prototype-2. The markers in Figure 10 show the magnetic flux density at the edges and the center of the ferrite. Simulation results demonstrated that the magnetic field distribution in the ferrite region reached sufficient uniformity.

**Figure 11:**
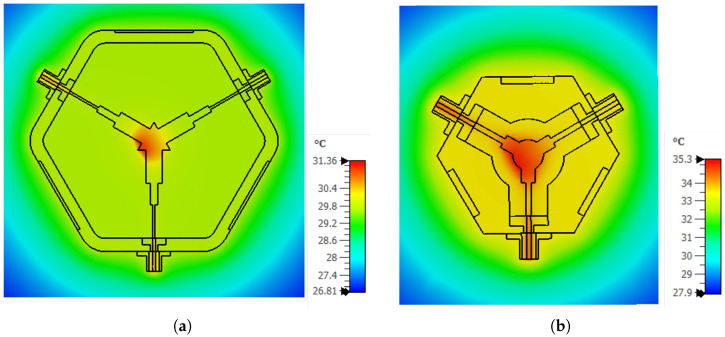
Thermal simulation results of the proposed stripline circulators: (**a**) Prototype-1 operating in the 2–4 GHz band, and (**b**) Prototype-2 operating in the 3–4 GHz band.

Following the magnetic field analyses, steady-state thermal simulations were performed for both proposed stripline circulator configurations to evaluate their thermal stability under high-power operating conditions. In these simulations, the electromagnetic loss distributions obtained from the frequency-domain solver were imported into the CST thermal solver and defined as volumetric heat sources. To represent the experimental high-power test conditions, a peak input power of 80 W, corresponding to an average power of approximately 40 W, was applied. The thermal material properties of the ferrite and dielectric layers, including thermal conductivity, density, and specific heat capacity, were assigned according to the parameters summarized in Table 9. Manufacturer-provided values were used where available, while the remaining parameters were selected within ranges widely reported in the literature to ensure physically realistic modeling. The ambient temperature was fixed at 25 °C. To accurately represent the experimental operating conditions, an open boundary condition was employed, and a convective heat transfer coefficient of 20 W/m^2^K was applied only to the surfaces exposed to the ambient environment. Under these assumptions, steady-state thermal simulations were carried out, and the resulting temperature distributions of the proposed circulator structures are presented in Figure 11.

For Prototype-1 (2–4 GHz, Figure 11a), the steady-state thermal solver predicts a maximum temperature of 31.36 °C and a minimum temperature of 26.81 °C. With an ambient temperature of 25 °C, the corresponding peak temperature rise is approximately ΔT≈6.4 °C. The hot spot is localized at the Y-junction center conductor region, coinciding with the area of maximum electromagnetic loss density. For Prototype-2 (3–4 GHz, Figure 11b), the maximum temperature reaches 35.29 °C (approximately 35.16 °C on the selected cross-section), while the minimum temperature is 27.92 °C, resulting in a peak temperature rise of ΔT ≈10.3°C. The elevated temperature level compared to Prototype-1 is consistent with the more compact geometry and increased loss concentration in the junction region. In both prototypes, the temperature distributions remain well within safe operating limits, and steady-state thermal equilibrium is achieved under natural air convection (h=20 W/m^2^K). These results are qualitatively consistent with the limited temperature rise observed during high-power experimental measurements.

**Figure 12:**
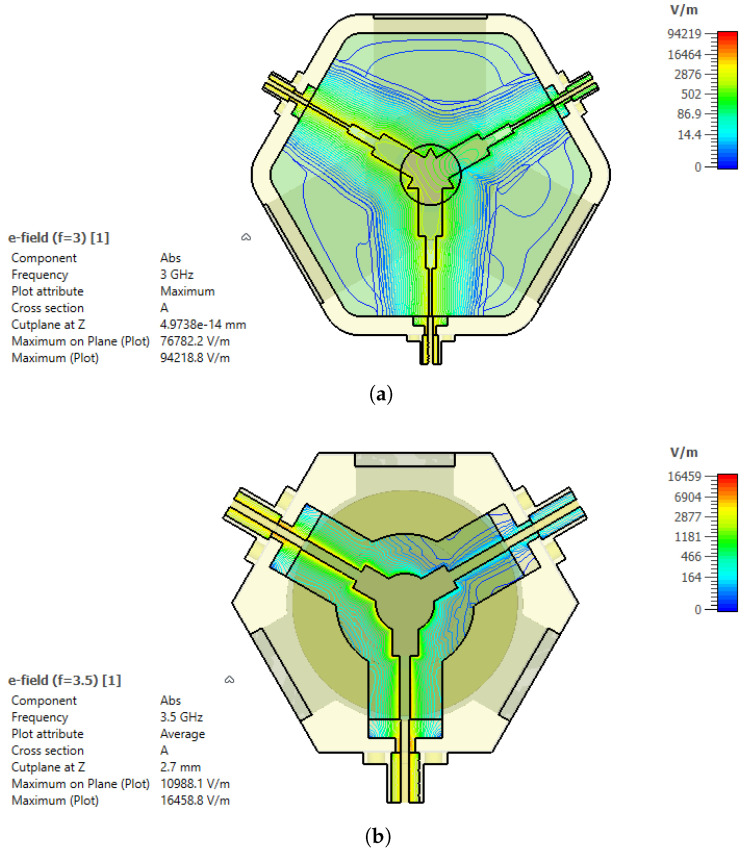
Simulated electric-field magnitude (|E|) distributions of the proposed stripline circulator: (**a**) 3 GHz, (**b**) 3.5 GHz.

In addition to the thermal analysis, the electric-field distribution was evaluated to assess the breakdown limitation under high-power operation. Figure 12 presents the simulated electric-field magnitude |E| at 3.5 GHz and 3 GHz. In both cases, the highest field intensities occur in the Y-junction center conductor region and at the SMA-to-stripline transition, where geometric discontinuities lead to field concentration. In linear passive microwave structures, the electric-field magnitude scales with the square root of the input power [1,13]. Therefore, the field level at an arbitrary input power *P* can be obtained from a reference solution computed at $P_{\mathrm{ref}}$ as

(14) $$E\left(P\right)=E_{\mathrm{ref}}\sqrt{\frac{P}{P_{\mathrm{ref}}}}.$$

The simulated fields were normalized to a reference input power of $P_{\mathrm{ref}}=0.5$ W using the frequency-domain solver [17]. Using the worst-case frequency point at 3 GHz, the maximum electric-field magnitude from the frequency-domain simulation is $E_{\max,\mathrm{ref}}=9.42\times 10^{4}$ V/m. When scaled to the applied peak input power of 80 W, the maximum field becomes

(15) $$E_{\max}\left(80\;W\right)=E_{\max,\mathrm{ref}}\sqrt{\frac{80}{0.5}}\approx 1.19\times 10^{6}\;V/m.$$

The air breakdown threshold was conservatively taken as $E_{\mathrm{bd},\mathrm{air}}\approx 3$ MV/m, yielding a safety margin

(16) $$M=\frac{E_{\mathrm{bd},\mathrm{air}}}{E_{\max}\left(80\;W\right)}\approx 2.5.$$

**Figure 13:**
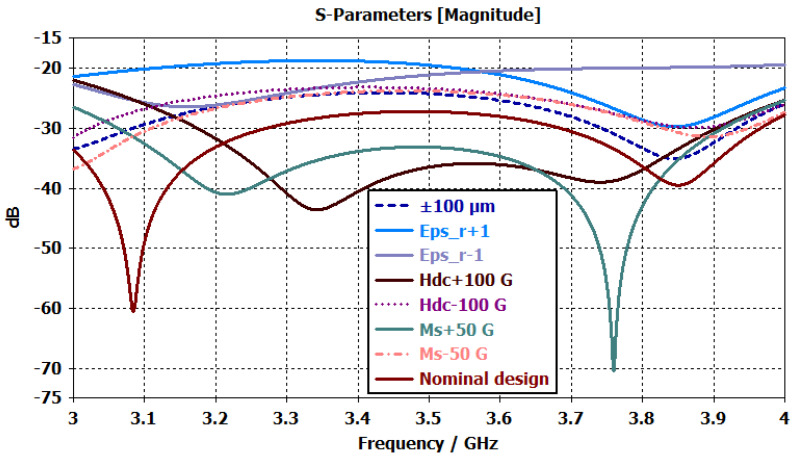
Sensitivity analysis of the input reflection coefficient ($S_{11}$) with respect to geometric tolerances, magnetic field variations, and material parameter uncertainties, compared to the nominal design.

Alternatively, the breakdown-limited peak input power can be estimated as

(17) $$P_{\mathrm{bd}}=P_{\mathrm{ref}}{\left(\frac{E_{\mathrm{bd},\mathrm{air}}}{E_{\max,\mathrm{ref}}}\right)}^{2}\approx 507\;W,$$

which is significantly higher than the applied power level. These results confirm that the proposed stripline circulator operates safely below the electric-field breakdown limit under the investigated high-power conditions. Before proceeding to the fabrication stage, the response of the proposed circulator designs to manufacturing tolerances, magnetic field variations, and uncertainties in material parameters was systematically evaluated. Magnetic field measurements performed on randomly selected samples from the same batch of permanent magnets revealed variations in the range of approximately 20–100G, despite identical nominal specifications. Based on this measurement range, the influence of magnetic field-related uncertainties on the circulator performance was investigated by varying the applied DC bias magnetic field ($H_{\mathrm{dc}}$) within ±100 G around the nominal operating point in the simulations. In addition, to assess the effects of manufacturing and material-induced uncertainties, all components were considered within the CST Microwave Studio environment. The geometric parameters of the ferrite disk, dielectric layers, and center conductor—including thickness, effective length, and characteristic radius (or equivalent line width)—were modeled using random variations within a range of ±100 µm to represent a realistic fabrication scenario. Furthermore, the relative permittivity of the dielectric material ($\varepsilon_{r}$) was varied by ±1, while the saturation magnetization of the ferrite material ($4\pi M_{s}$) was adjusted within ±50G and incorporated into the tolerance analyses. The results of the tolerance analyses are presented in Figure 13. The figure illustrates the variations in the input return loss characteristics of the circulator under geometric tolerances (±100 µm), changes in dielectric permittivity ($\varepsilon_{r}\pm 1$), deviations in the DC bias magnetic field ($H_{\mathrm{dc}}\pm 100\;G$), and variations in ferrite saturation magnetization ($4\pi M_{s}\pm 50\;G$). The results indicate that variations in the dielectric permittivity have a pronounced impact on the return loss level across the operating band, whereas changes in the magnetic field and saturation magnetization primarily lead to localized shifts in resonance depth and frequency. In contrast, the influence of geometric tolerances remains comparatively limited with respect to the nominal design.

**Figure 14:**
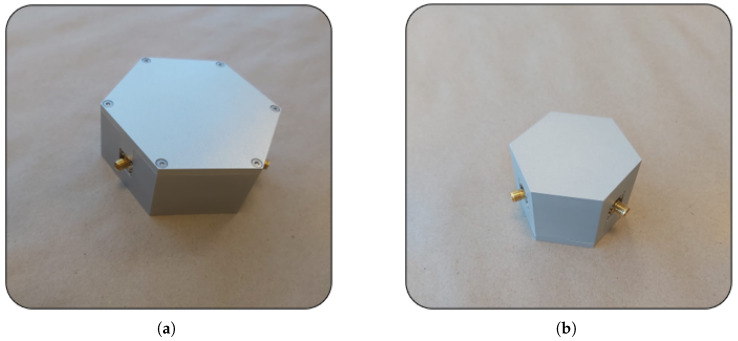
Assembled views of the stripline circulators: (**a**) 2–4 GHz, and (**b**) 3–4 GHz.

After the targeted performance values were achieved in the simulation stage, three-dimensional solid models were prepared for both circulators, and the fabrication process was initiated. In this context, the body structures were machined with high precision on CNC machines in accordance with the ISO 2768 tolerance standard [23] and the center conductor geometries were manufactured by wire EDM to satisfy the strict tolerance requirements. Assembly is a critical stage that directly influences device performance. For this reason, all components were mechanically fixed and precisely aligned to maintain structural symmetry. The center conductor and connector inputs were carefully soldered to ensure both mechanical integrity and electrical conductivity. In addition, the magnetic circuit elements were positioned accurately using mechanical fasteners. The assembled views of the circulators are shown in Figure 14.

## 4. Measurement of Stripline Circulators

After assembly, a three-stage testing procedure was performed to investigate the transmission, isolation, and thermal behavior of the circulators. In the first stage, the magnetic field distributions generated by the permanent magnets were mapped to assess field homogeneity on the ferrite disk and to verify the conditions required for directional transmission. In the second stage, low-power measurements were performed under a constant magnetic field using a vector network analyzer (VNA) to determine return loss, insertion loss, and isolation across the operating band. In the final stage, high-power tests were performed to evaluate the impact of temperature rise on transmission, thereby assessing the overall performance of the devices.

### 4.1. Magnetic Field Distribution Measurement

**Figure 15:**
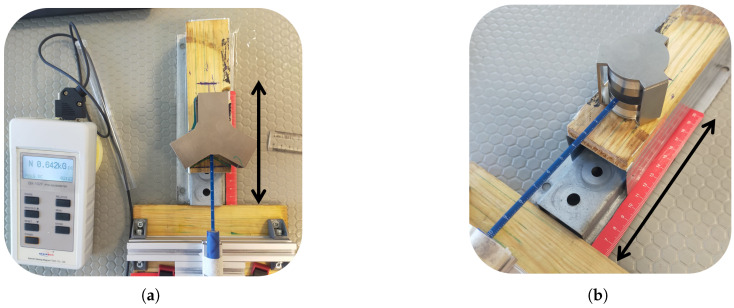
Experimental magnetic field measurement setup for the stripline circulators: (**a**) 2–4 GHz configuration, (**b**) 3–4 GHz configuration.

The magnetic field distribution produced by permanent magnets on the ferrite disk was measured in detail, as it directly influences the directional transmission performance of the device. Manual scans were performed along the Y-axis at 1 mm intervals to evaluate field uniformity, and the effect of different magnet thicknesses was systematically investigated. All magnets used in the tests were N35 grade with a diameter of 20 mm, arranged in combinations of 1 mm, 2 mm, 2.5 mm, 3 mm, and 5 mm thicknesses. The measurement setup consisted of magnet slots, magnetic field flattening plates, and a magnetic flux return yoke (Figure 15). Field measurements were carried out using a DX-102F Gaussmeter [24] with a Hall sensor and a manually controlled scanning setup.

**Figure 16:**
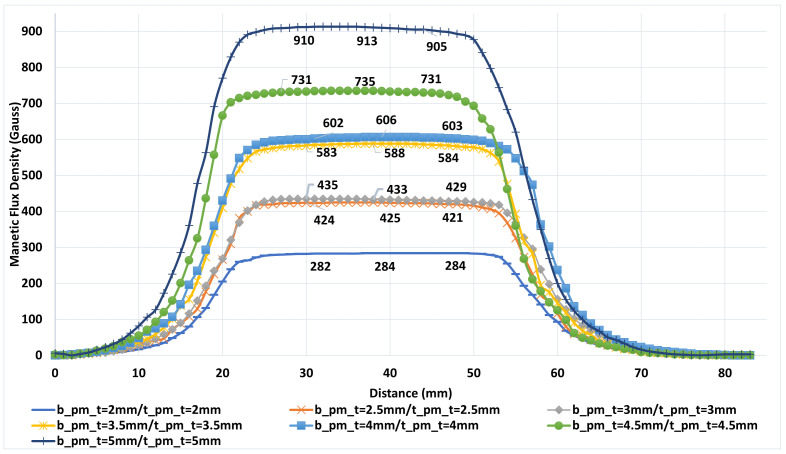
Measured magnetic flux density distribution of the 2–4 GHz stripline circulator.

**Figure 17:**
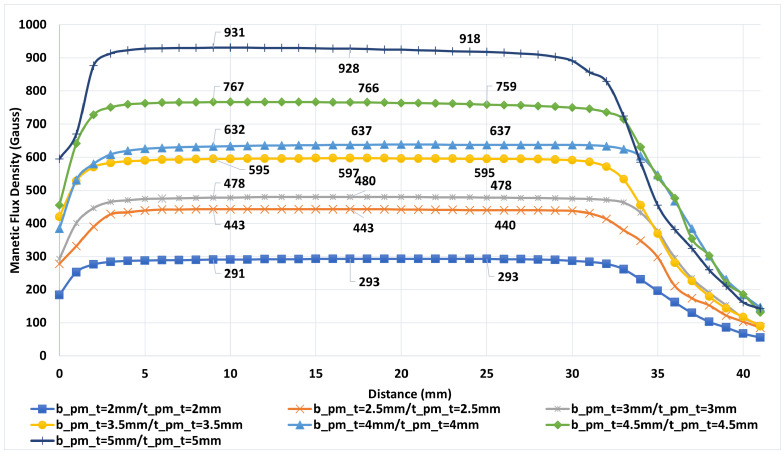
Measured magnetic flux density distribution of the 3–4 GHz stripline circulator.

The labels in Figure 16 and Figure 17 indicate the thickness configurations of the magnet pairs. In this notation, $b_{{\mathrm{pm}}_{t}}$ refers to the thickness of the bottom magnet, whereas $t_{{\mathrm{pm}}_{t}}$ denotes that of the top magnet. Measurements across these configurations revealed a uniform magnetic field profile in the center region containing the ferrite disk. The maximum field variation observed at the labeled points was about ±10 Gauss. Minor deviations appeared at certain data points due to positioning errors in manual scanning; however, these did not affect the overall homogeneity of the magnetic field. As shown in Figure 16 and Figure 17, the limited physical dimensions of the magnetic circuit used in prototype-2 restricted the measurement area. Although this limitation does not capture the complete magnetic field profile, it provides sufficient accuracy for characterizing the effective field intensity over the ferrite region.

When the simulation and measurement results were compared, noticeable differences were observed for the 5 mm thick magnet between CST EM predictions and experimental data. In particular, the measured field intensity in the center region was lower than the simulated values. This deviation can be attributed to the actual remanence of the magnets being lower than the nominal values specified by the manufacturer.

### 4.2. Scattering Parameter Measurement with VNA

**Figure 18:**
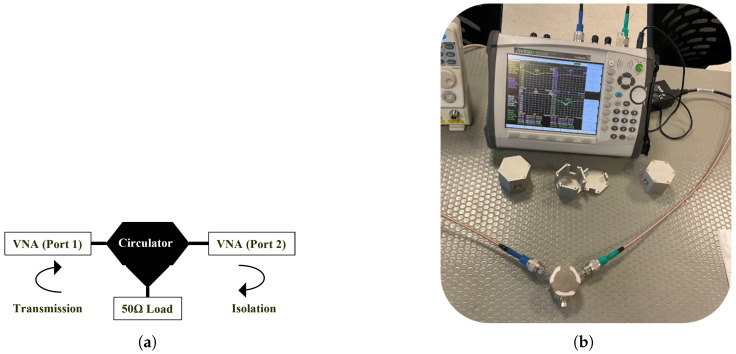
Low-power measurement setup of the stripline circulator: (**a**) schematic diagram of the transmission and isolation measurement, (**b**) experimental setup used with the VNA.

Under constant magnetic field conditions, the transmission ($S_{21}$), return loss ($S_{11}$), and isolation ($S_{31}$) parameters of the circulators were measured using a vector network analyzer (VNA), thereby enabling the evaluation of their in-band performance. Measurements were performed for two different ferrite groups: locally manufactured ferrite disks (Solak Lab.) and commercial AL800 ferrites (TCI). For each group, the scattering parameters were obtained under magnetic field configurations generated by N35 permanent magnets with thickness combinations of 1 mm, 2 mm, 2.5 mm, 3 mm, and 5 mm. The experiments were carried out using an Anritsu MS2026C vector [25] network analyzer, two N-type coaxial cables, a 50 Ω SMA dummy load, and N–SMA adapters. During the test, the third port was terminated with a matched load, while the other two ports were connected to the VNA. The complete measurement setup is shown in Figure 18.

Furthermore, for the experimental measurements, Solak Laboratory fabricated five hexagonal and six Y-shaped dielectric substrates. These substrates were used to assemble circulator prototypes employing both locally produced and commercial ferrites, and their transmission and isolation characteristics were measured under different magnetic field levels.

**Figure 19:**
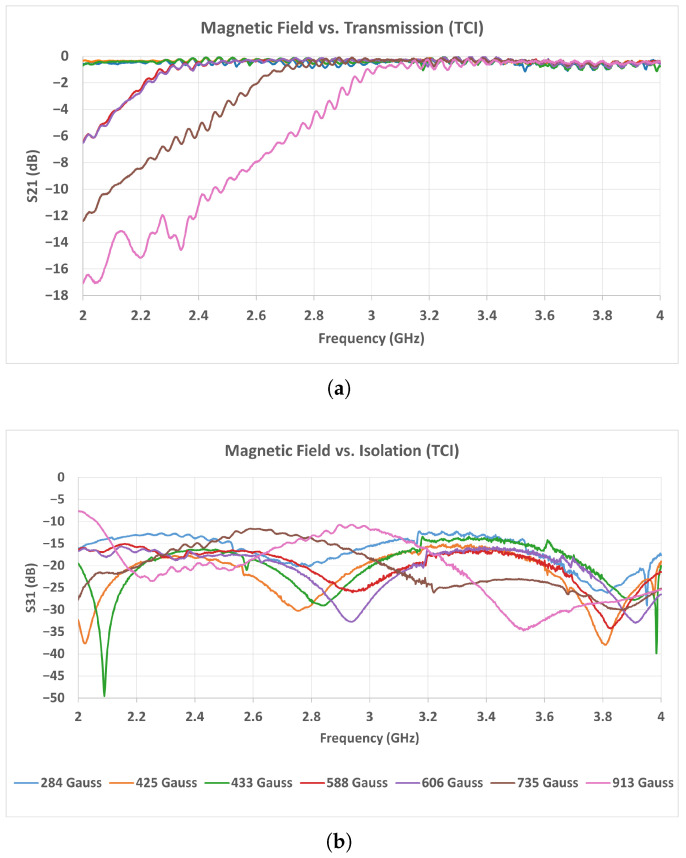
Measured scattering parameters of the 2–4 GHz circulator as a function of magnetic field intensity using TCI ferrite: (**a**) $S_{21}$, (**b**) $S_{31}$.

**Figure 20:**
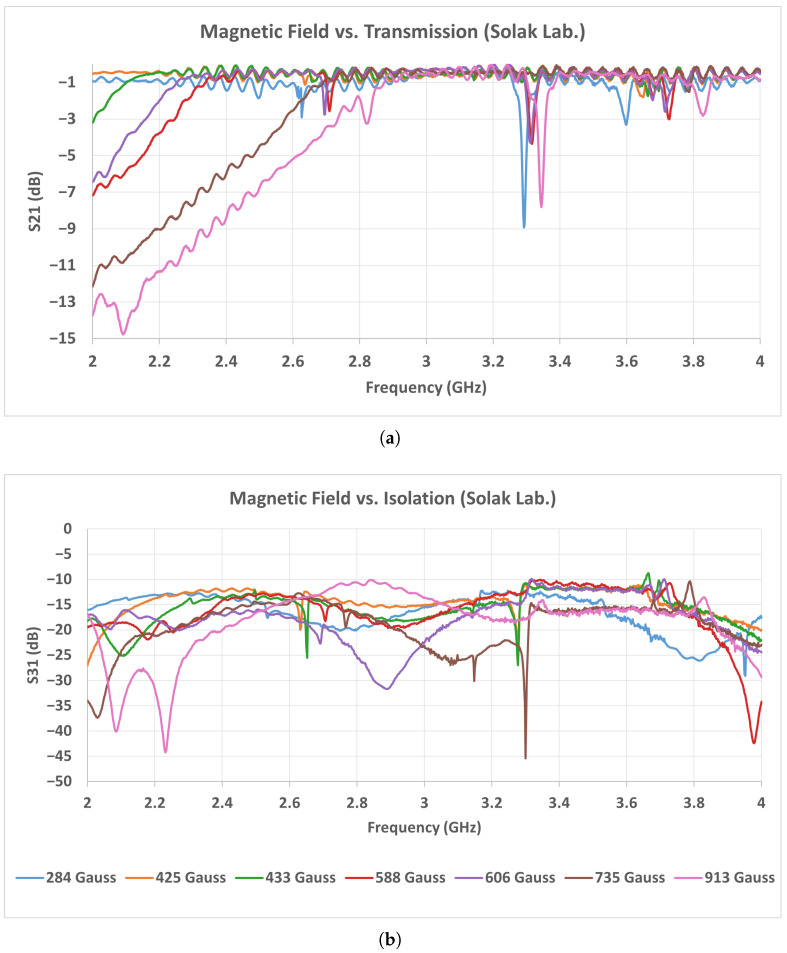
Measured scattering parameters of the 2–4 GHz circulator as a function of magnetic field intensity using Solak Lab. ferrite: (**a**) $S_{21}$, (**b**) $S_{31}$.

As seen in the measurement results of Figure 19 and Figure 20, the TCI and Solak ferrites exhibit similar transmission behavior in the low magnetic field range between 284 and 433 Gauss. Within this range, both ferrites maintain insertion loss values typically between 0.1 and 0.5 dB over the entire operating band, indicating stable low-loss operation. In this interval, no significant resonance dips or instability are observed in either ferrite type. When the magnetic field exceeds approximately 450 Gauss, the performance of the Solak ferrite begins to degrade. In this region, the transmission level decreases noticeably, particularly in the 2.2–2.8 GHz frequency range, and the insertion loss increases accordingly. The degradation becomes more pronounced as the magnetic field is further increased, leading to a less uniform transmission response across the band. In contrast, the TCI ferrite preserves a smoother and more consistent transmission profile at higher magnetic field levels, with fewer amplitude variations and no strong localized loss regions. These results indicate that the Solak ferrite is more sensitive to higher magnetic field values, which results in increased loss and reduced transmission stability under these conditions.

**Figure 21:**
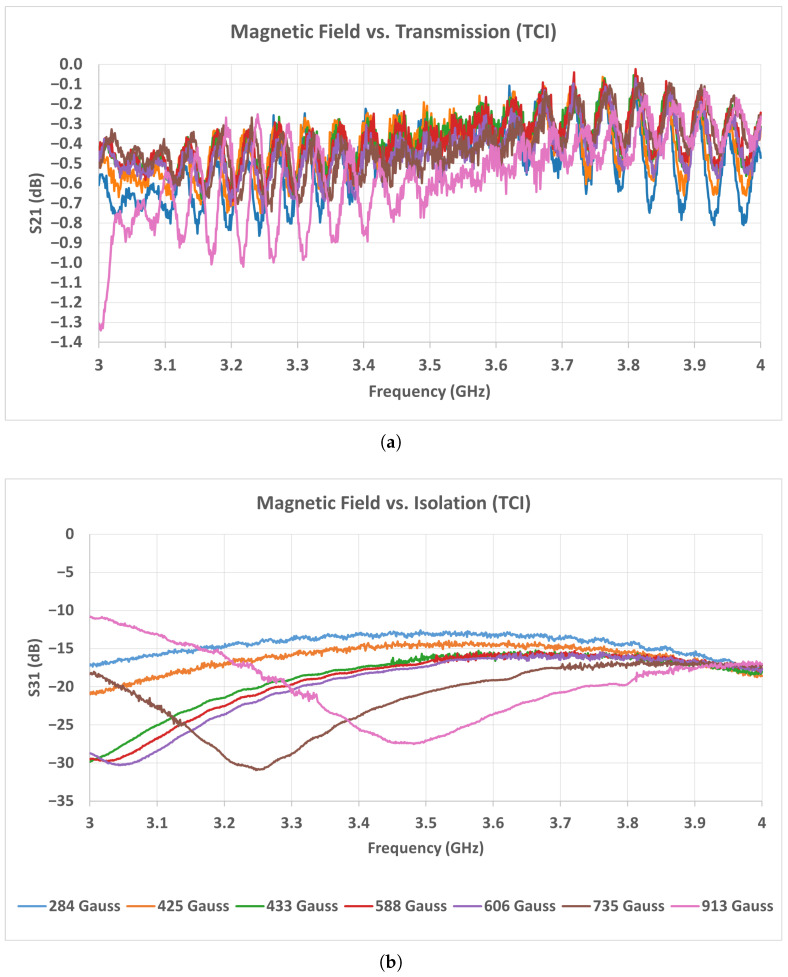
Measured scattering parameters of the 3–4 GHz stripline circulator as a function of magnetic field intensity using TCI ferrite: (**a**) $S_{21}$, (**b**) $S_{31}$.

**Figure 22:**
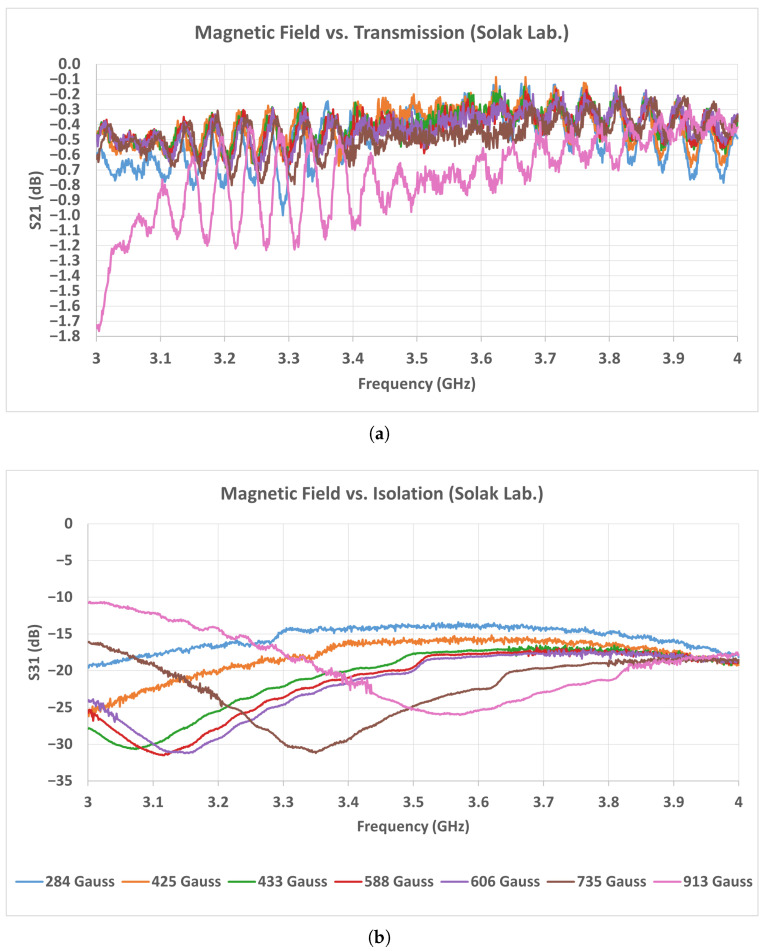
Measured scattering parameters of the 3–4 GHz stripline circulator as a function of magnetic field intensity using Solak Lab. ferrite: (**a**) $S_{21}$, (**b**) $S_{31}$.

A similar trend is observed in the 3–4 GHz measurement results shown in Figure 21 and Figure 22. In this band, the TCI ferrite maintains a stable insertion loss behavior as the magnetic field increases, preserving low-loss performance across the entire frequency range. The transmission response remains smooth, and no strong degradation is observed within the investigated magnetic field interval. For the Solak ferrite, the lowest insertion loss values are obtained in the magnetic field range between 425 and 700 Gauss. Within this interval, the transmission remains acceptable over most of the band. However, when the magnetic flux density exceeds approximately 750 Gauss, the transmission degrades rapidly. In this case, broader and deeper loss regions appear across the band, leading to a clear increase in insertion loss. Considering the results obtained in both frequency bands, the Solak ferrite exhibits a narrower magnetic field range where acceptable performance is achieved. Outside this range, the insertion loss increases noticeably. In contrast, the TCI ferrite provides stable and low-loss operation over a wider magnetic field interval in both the 2–4 GHz and 3–4 GHz bands.

**Figure 23:**
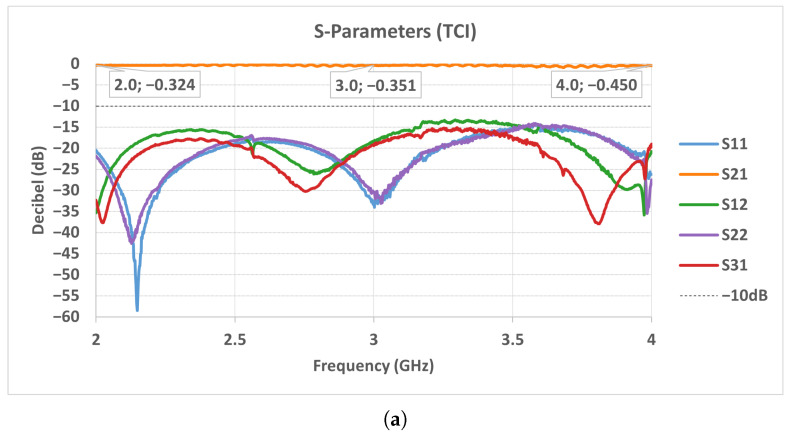

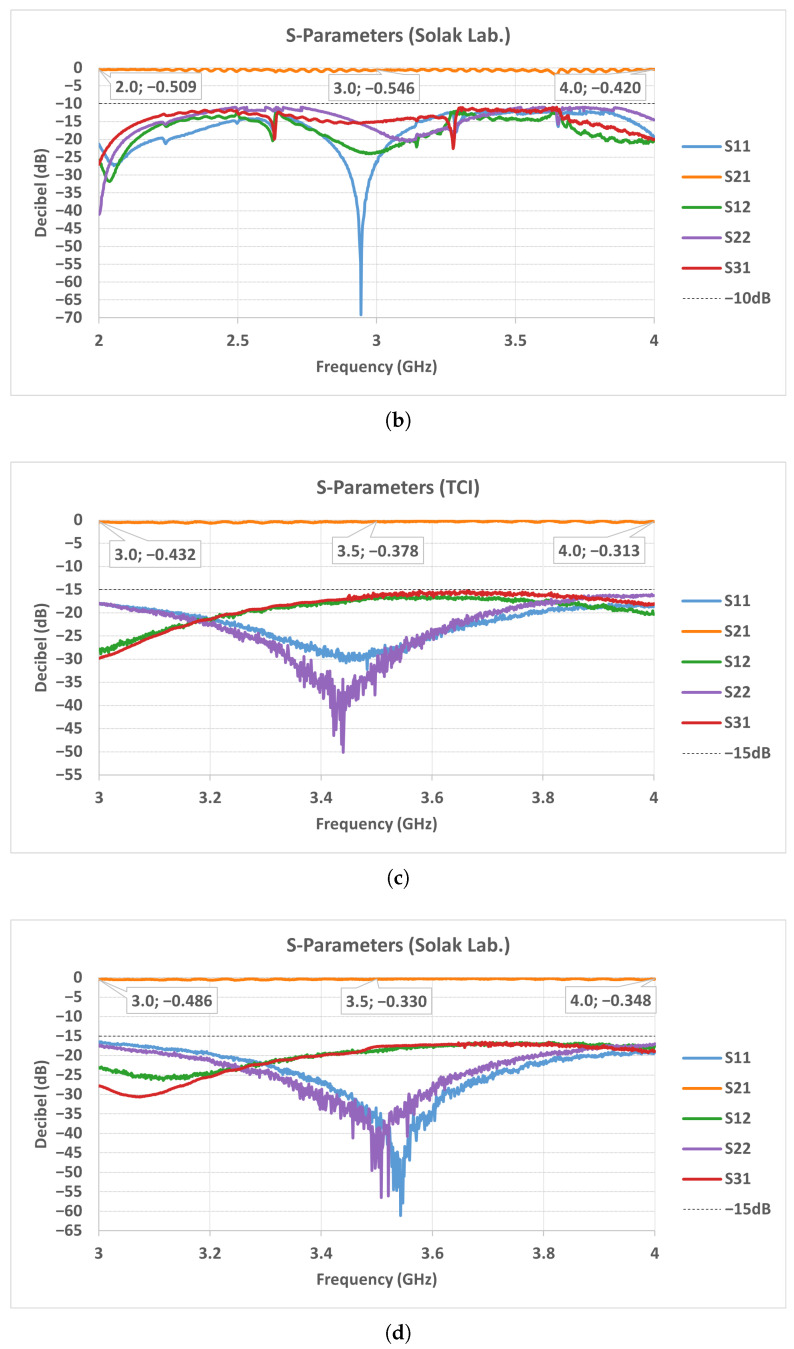
Measured scattering parameters of the stripline circulators: (**a**) 2–4 GHz (TCI), (**b**) 2–4 GHz (Solak Lab.), (**c**) 3–4 GHz (TCI), (**d**) 3–4 GHz (Solak Lab.).

The lowest insertion-loss values were obtained within the 425–700 Gauss interval, where both prototypes exhibited their most stable transmission characteristics. Based on this observation, the optimum magnetic field levels were set to 425 Gauss for Prototype-1 (2–4 GHz) and 433 Gauss for Prototype-2 (3–4 GHz). Under these optimized bias conditions, the measured scattering parameters are presented in Figure 23. The S-parameter curves confirm that both circulators achieve low insertion loss, high isolation, and good port matching across their respective operating bands, with the TCI ferrites showing a smoother and more uniform response compared to the locally produced ferrites.

### 4.3. High-Power and Thermal Tests

In the final stage, thermal and transmission stability tests of the circulators were conducted at a center frequency of 2.995 GHz, considering the frequency limitations of the spectrum analyzer, to assess their performance under high-power operation. The experiments employed a GW Instek spectrum analyzer covering the 9 kHz–3 GHz frequency range. Prior to these tests, the S-parameter characteristics of the circulators at 2.995 GHz were measured using an Anritsu MS2026C vector network analyzer (VNA), and their low-power transmission profiles were recorded (Table 10).

**Figure 24:**
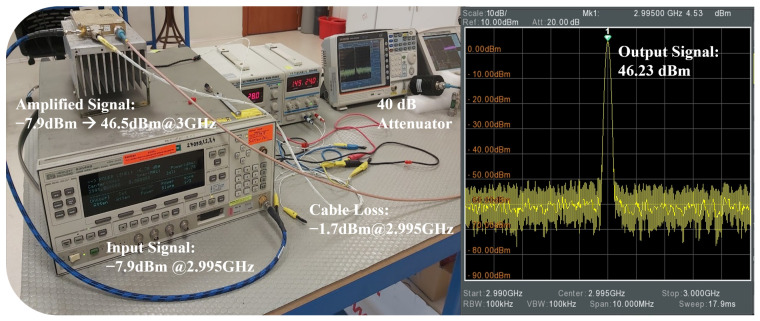
Measurement setup used to determine the input–output power characteristics of the circulator.

Subsequently, a continuous RF signal at 2.995 GHz was generated using a signal generator and fed to the spectrum analyzer through a 40 dB attenuator, employing the measurement setup shown in Figure 24. The signal was then amplified by an RF power amplifier in the transmission line, and both input and output power levels were measured separately. The attenuation of the RF cables in the setup was also quantified and incorporated into the overall gain/loss profile. Taking into account the cable loss (1.7 dB) and the attenuator loss (40 dB), the effective output power was calculated as 46.23 dBm (41.976 W).

**Figure 25:**
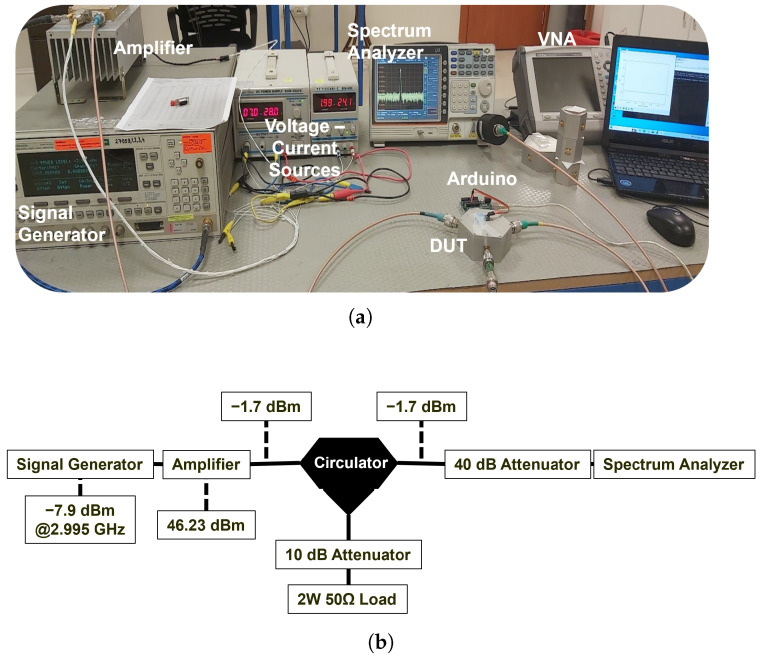
Experimental for circulator power measurements: (**a**) photograph of the measurement setup, (**b**) schematic representation of the power measurement configuration.

As illustrated in Figure 25, each circulator was continuously subjected to the predetermined maximum input power level for a duration of 15 min. Throughout the test, device temperatures were monitored and recorded at one-minute intervals, and any variations in the transmission parameters were also registered.

**Figure 26:**
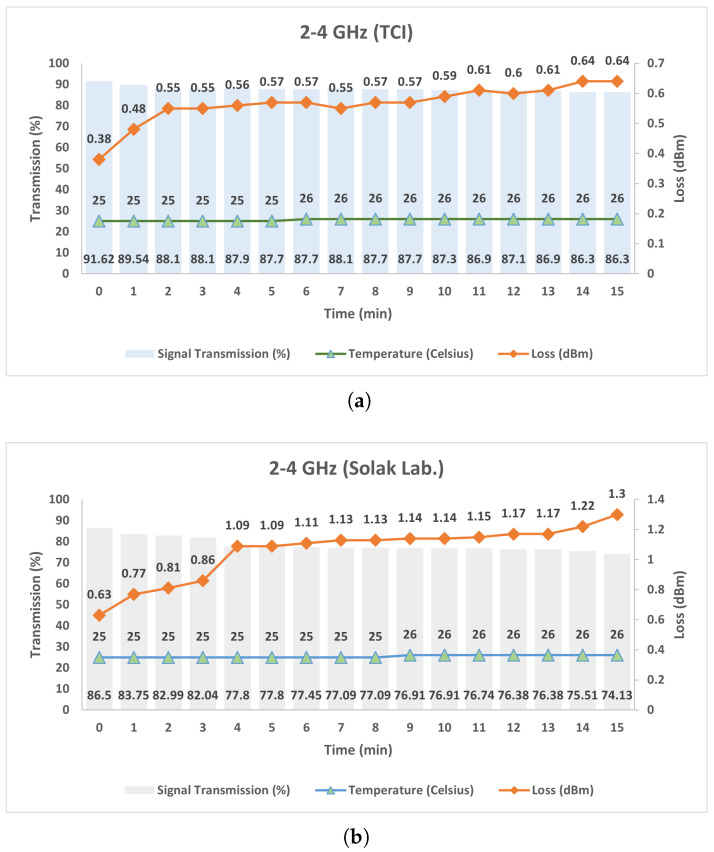
Measured insertion loss and transmitted power of the stripline circulators under high-power operation as a function of time: (**a**) 2–4 GHz (TCI), (**b**) 2–4 GHz (Solak Lab.).

In Figure 26a,b, the temperature (°C), insertion loss (dB), and transmission ratio (%) obtained during high-power tests of Prototype-1 using TCI and Solak ferrites are presented as a function of time. The circulator employing TCI ferrite exhibited highly stable thermal behavior during the test, with the temperature remaining within 25–26 °C. Meanwhile, the transmission ratio gradually decreased from 91.6% to 86.3%, and the insertion loss increased from 0.38 dB to 0.64 dB. These results indicate that the device operated in a thermally stable manner under high-power excitation while largely preserving its electromagnetic performance. In contrast, the circulator fabricated with Solak ferrite exhibited more pronounced electromagnetic performance degradation under the same operating conditions. The transmission ratio decreased from 86.5% to 74.1%, and the insertion loss increased from 0.63 dB to 1.30 dB, although the temperature profile remained stable within the 25–26 °C range. These observations indicate that, while both configurations are thermally stable, the TCI ferrite provides improved RF performance stability for Prototype-1 under prolonged high-power operation.

**Figure 27:**
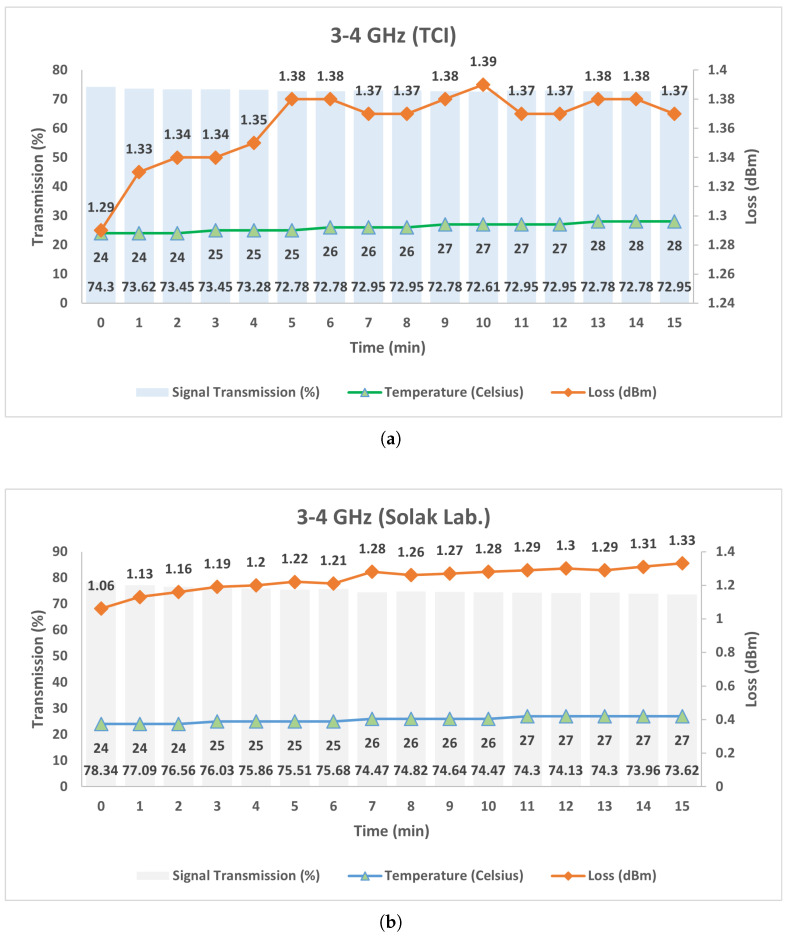
Measured insertion loss and transmitted power of the stripline circulators under high-power operation as a function of time: (**a**) 3–4 GHz (TCI), (**b**) 3–4 GHz (Solak Lab.).

According to the results presented in Figure 27a,b, when the Prototype-2 circulator employing TCI ferrite was subjected to a continuous high-power signal for 15 min, the transmission ratio decreased slightly from 74.3% to 72.95%, corresponding to a total reduction of only 1.35%. During the same interval, the insertion loss increased marginally from 1.29 dB to a maximum value of 1.39 dB. The temperature increased gradually from 24 °C to 28 °C, confirming thermally stable operation without critical overheating. For the circulator employing Solak ferrite, the temperature rise remained similarly limited (24–27 °C); however, the transmission ratio exhibited a larger decrease and the insertion loss increased more noticeably compared to the TCI ferrite case. Overall, both Prototype-2 configurations maintained thermal stability under high-power operation, while the TCI ferrite demonstrated superior electromagnetic performance stability.

### 4.4. Comparison with Previously Reported Wideband Stripline Circulators

A quantitative comparison between the proposed designs and representative stripline circulators reported in the literature is presented in Table 11. As shown in the table, Prototype-1 and Prototype-2 achieve low insertion loss (<0.45 dB and <0.43 dB), sufficient isolation (>15 dB and >16 dB), and good impedance matching across their respective operating bands, demonstrating performance levels comparable to well-established broadband designs such as [8]. Beyond the numerical S-parameter values summarized in Table 11, the proposed circulators are distinguished by the extent of experimental validation. While several reported studies rely primarily on VNA-based measurements (e.g., [9,10]), the present work incorporates magnetic field mapping, steady-state thermal and electric-field analyses, and continuous-wave high-power measurements, enabling a more realistic assessment of device behavior under practical operating conditions. The wideband design reported in [13] achieves a broader fractional bandwidth; however, its power-handling capability is mainly demonstrated through numerical simulations. In contrast, the high-power performance of the proposed prototypes is verified experimentally at the hardware level and supported by complementary simulation results. In addition, the inclusion of both locally manufactured and commercial ferrite materials under identical conditions provides practical insight into material-dependent performance variations. Overall, the comparison in Table 11 indicates that competitive broadband S-band circulator performance can be achieved using a unified design approach combined with systematic measurement and characterization.

## 5. Conclusions

In this study, two broadband stripline circulators operating in the 2–4 GHz and 3–4 GHz frequency bands were designed, fabricated, and experimentally characterized using the same ferrite type and coupling angle. Both prototypes satisfied the targeted specifications under low- and high-power operating conditions. The 2–4 GHz prototype achieved transmission levels close to 90% with a maximum loss of 0.45 dB and isolation better than 15 dB. The 3–4 GHz prototype also demonstrated broadband operation, although its performance was more sensitive to impedance matching limitations. High-power tests conducted at an input power level of 40 W showed that the circulator employing TCI ferrite maintained stable transmission characteristics, with a limited temperature rise on the order of 2–4 °C and a maximum loss of 0.64 dB. In contrast, the circulator fabricated with locally produced ferrite exhibited reduced electromagnetic stability under the same high-power conditions, with the transmission ratio decreasing to approximately 74% and the loss increasing to about 1.3 dB. Magnetic field measurements confirmed a sufficiently uniform bias field distribution, with deviations remaining within ±10 Gauss, ensuring effective circulation behavior. Overall, the results demonstrate that broadband operation over multiple frequency bands can be achieved using a common ferrite type and design geometry. In addition, the comparative evaluation offers valuable feedback to local ferrite manufacturers, providing practical guidance for the future optimization of domestically produced ferrite materials. 

## Figures and Tables

**Table 1:** Target design parameters of the circulator prototypes.

Parameter	Prototype-1	Prototype-2
Frequency Range (GHz)	2–4	3–4
Insertion Loss (dB)	<0.5	<0.5
Isolation (dB)	>14	>14
Transmission (%)	90	95
Average Power (W)	35	35
Peak Power (W)	50	50

**Table 2:** Saturation magnetization ranges calculated at center frequencies.

Center Frequency (MHz)	Saturation Magnetization $4\pi M_{s}$ (Gauss)		Condition
	Minimum	Maximum	
3000	357.1	803.6	–
3500	416.7	937.5	–
3000	535.7	857.1	$H_{a}=0$
3500	625.0	1000.0	$H_{a}=0$
3000	435.1	757.1	$H_{a}=100$
3500	525.0	900.0	$H_{a}=100$

**Table 3:** Properties of the AL800 ferrite material [18].

Parameter	Value
Saturation Magnetization	800 Gauss
Linewidth	30 Oe
Curie Temperature	199 °C
Relative Permittivity	14.6
Dielectric Loss Tangent	<0.0002
Lande Factor	2.01
Product Code	AL800

**Table 4:** Ferrite disk radius values calculated at different center frequencies for $4\pi M_{s}=800\;G$, $\varepsilon_{r}=14.6$ and kR=1.84.

*f* (MHz)	κ/μ	$\mu_{\mathrm{eff}}$	$\lambda_{0}$ (mm)	$R_{f}$ (mm)
3000	0.75	0.44	99.93	11.5
3150	0.71	0.49	95.17	10.4
3500	0.64	0.60	85.65	8.5
3675	0.61	0.63	81.58	7.9

**Table 5:** Calculated network parameters.

*f* (MHz)	${\mathrm{VSWR}}_{\min-\max}$	$Y_{\mathrm{eff}}$	$Q_{L}$	$Q_{u}$	IL (dB)	$G_{r}$ (S)	*d* (mm)	$Z_{T1}$(Ω)	$Z_{T2}$(Ω)
3000	1.05–1.20	0.0152	0.6793	93.87	0.0315	0.2166	9.75	9.50	32.03
3150	1.05–1.20	0.0144	0.6793	103.29	0.0287	0.2166	8.30	9.50	32.03
3500	1.05–1.11	0.0132	1.442	126.90	0.0496	0.0765	2.786	24.27	–
3675	1.05–1.11	0.0128	1.442	139.55	0.0451	0.0765	2.500	24.27	–

**Table 6:** Analytically calculated parameters for the designed circulators.

Parameters	Prototype-1	Prototype-2
$R_{f}$ (mm)	11.5	8.5
$R_{c}$ (mm)	7.65	4.7
*d* (mm)	9.75	2.8
*W* (mm)	6.85	4.2
sinψ (rad)	0.447	0.447
$Z_{T1}$ (Ω)	9.5	24.27
$Z_{T2}$ (Ω)	32.03	–
$L_{T1,2}$ (mm)	8.33	7.15

**Table 7:** Comparison of analytically calculated and CST optimized parameters.

Parameters	Prototype-1		Prototype-2	
	Analytical	Simulated	Analytical	Simulated
$R_{f}$ (mm)	11.5	7.95	8.5	7.95
$R_{c}$ (mm)	7.65	7.1	4.7	4.4
$R_{d}$ (mm)	–	–	$1.3R_{f}$	10.35
*d* (mm)	9.75	2.55	2.8	2.55
*W* (mm)	6.85	6.3	4.2	3.9
sinψ (rad)	0.447	0.4436	0.447	0.4432
$Q_{L}$	0.679	–	1.44	–
$Z_{r}=1/G_{r}$ (Ω)	13.85	–	13.08	–
$Z_{T1}$ (Ω)	9.5	17.82	25.5	37.34
$Z_{T2}$ (Ω)	32.03	29.73	–	–
$Z_{T3}$ (Ω)	–	45.8	–	–
$L_{T1,2,3}$ (mm)	8.33	$L_{T1,2}=8.3$, $L_{T3}=12.7$	7.14	$L_{T}=7.15+2.4$

**Table 8:** Properties of the permanent magnet used in the circulator designs.

Parameter	Value
Remanent Magnetization	1.2 Tesla
Magnet Diameter	20 mm
Magnet Thickness	5 mm
Product Code	N35 (Neodymium)

**Table 9:** Thermal material parameters used in the simulations.

Material	Parameter	Value	Unit	Source
K9	Thermal conductivity (*k*)	18.8	W/m·K	[18]
	Linear thermal expansion (α)	$115\times 10^{-6}$	$K^{-1}$	[18]
	Density (ρ)	2800	kg/m^3^	[18]
	Specific heat ($c_{p}$)	800	J/kg·K	[18]
AL800	Thermal conductivity (*k*)	4.2–5.4	W/m·K	[21]
	Linear thermal expansion (α)	(8.0–$10.5)\times 10^{-6}$	$K^{-1}$	[21]
	Density (ρ)	5000–5200	kg/m^3^	[22]
	Specific heat ($c_{p}$)	400–500	J/kg·K	[22]

**Table 10:** Measured scattering parameters of the circulators at 2.995 GHz.

Parameter	Prototype-1 (TCI)	Prototype-1 (Solak Lab.)	Prototype-2 (TCI)	Prototype-2 (Solak Lab.)
$S_{11}$ (dB)	−28.28	−28.33	−19.01	−17.11
$S_{12}$ (dB)	−18.29	−24.29	−26.65	−25.68
$S_{21}$ (dB)	−0.27	−0.66	−0.50	−0.55
$S_{22}$ (dB)	−28.21	−17.88	−17.57	−18.01

**Table 11:** Comparison of the proposed stripline circulators with representative designs reported in the literature.

Reference	Band (GHz)	BW (%)	IL (dB)	ISO (dB)	RL/VSWR
This work (P1)	2–4	66.7 (1 octave)	<0.45	>15	RL > 15 dB
This work (P2)	3–4	28.6	<0.43	>16	RL > 17 dB
[8]	2.6–5.2	66.7	<0.40	>20	VSWR ≤ 1.20
[13]	6–18	100	<2	<12	RL > 15 dB
[13]	1.4–1.6	13.4	1.5	19.4	RL > 25.3 dB
[9]	3.3–4.3	26	<0.36	>23	RL > 20 dB
[10]	10.5–13.5	25	<0.19	>25	RL > 23 dB
[26]	2–4	66.7	0.5	18	VSWR ≤ 1.30

## Data Availability

The data presented in this study are available on request from the corresponding author.

## Abbreviations

The following abbreviations are used in this manuscript:

AL800	Aluminum-doped ferrite material
AR	Above-resonance mode
BR	Below-resonance mode
CST	Computer Simulation Technology
GHz	Gigahertz
HFSS	High-Frequency Structure Simulator
IL	Insertion Loss
K9	Dielectric material surrounding the ferrite
MHz	Megahertz
N35	Neodymium magnet grade
Oe	Oersted
RF	Radio Frequency
SMA	SubMiniature Version-A connector
S-parameters	Scattering parameters
Solak Lab.	Local manufacturer of ferrite and dielectric materials (Kupfer Advanced Materials
	Technologies, Turkey)
TCI	Ferrite manufacturer (TCI Ceramics, National Magnetics Group, Inc., USA)
VNA	Vector Network Analyzer
VSWR	Voltage Standing Wave Ratio
YIG	Yttrium Iron Garnet
YG	Yttrium Gadolinium Garnet
Y-junction	Three-port circulator junction
